# Inter-Population Variability of Terpenoid Composition in Leaves of *Pistacia lentiscus* L. from Algeria: A Chemoecological Approach

**DOI:** 10.3390/molecules16032646

**Published:** 2011-03-22

**Authors:** Samir Ait Said, Catherine Fernandez, Stéphane Greff, Franck Torre, Arezki Derridj, Thierry Gauquelin, Jean-Philippe Mevy

**Affiliations:** 1 Faculté des Sciences Biologiques et des Sciences Agronomiques, Université Mouloud MAMMERI BP 17 Tizi-Ouzou, Algeria; 2 Institut Méditerranéen d’Ecologie et Paléoécologie (IMEP), UMR CNRS 6116, Equipe Diversité Fonctionnelle des Communautés Végétales, Aix-Marseille Université, Centre Saint Charles. 3, place Victor Hugo. 13331, Marseille Cedex 3, France; 3 Institut Méditerranéen d’Ecologie et Paléoécologie (IMEP), UMR CNRS 6116, Equipe Population, Communauté, Paysage. case 461, 13397, Aix-Marseille Université, Marseille Cedex 20, France

**Keywords:** *Pistacia lentiscus*, terpenes, altitude, Algeria, α-pinene, β-caryophyllene, caryophyllene oxide, cubebol, β-bisabolene, variability

## Abstract

Three different altitudes were selected to study the variability of terpenoid composition from leaves of female plants of *Pistacia lentiscus* L. throughout the elevation gradient. GC-MS analyses showed that terpenoid contents change with altitude. Forty nine compounds were identified with a high interpopulation variability for low- and midaltitude sites that also exhibited the same major components when data were expressed on dry weight basis. However, Two-Way-ANOVA followed by Tukey’s *post hoc* test showed that monoterpene hydrocarbons increased with elevation, giving values of 21.7, 37.5 and 221.5 µg g^−1^ dw for low- mid- and highlands, respectively. On the other hand, applying P.C.A. with data expressed in percentage of the chromatogram of the volatile extract led to the identification of three chemotypes associated with altitudinal levels. In highlands (Group I), the major compounds were β-caryophyllene (12%), δ-cadinene (9.3%) and α-pinene (6.3%) while in midlands (Group II), β-caryophyllene (11.5%), δ-cadinene (8.6%) and caryophyllene oxide (6.8%) were the main components. In lowlands (Group III) δ-cadinene (10.9%), cubebol (10.5%) and β-bisabolene (7.7%) were chiefly present. Hence, the involvement of genetic factors, temperature and drought in the chemical polymorphism of *P. lentiscus* associated with elevation is discussed in this report.

## 1. Introduction

Biosynthesis of secondary metabolites is not only controlled genetically, but it is also strongly affected by different biotic and abiotic stresses [1]. Among plant secondary metabolites, terpenoids are the most abundant and structurally diverse group [2]. 

Altitude is one of the abiotic stresses associated with alterations in a number of environmental factors such as air temperature, precipitation, wind exposure, light intensity, UV-B radiation, ozone density and oxidizing air pollutants [3]. The effect of the altitudinal gradient on essential oil content from many species has been evaluated by several authors [4,5]. 

*Pistacia lentiscus* L. (Anacardiaceae) is a sclerophyllous dioecious shrub which forms bushes of up to 2 m height, sometimes attaining a tree growth form in more humid and protected sites [6]. It is a low altitude species [7], which has been found to be one of the more drought tolerant plants among other evergreen species [8] and to be very tolerant to salinity [9]. This species is very common in the Mediterranean Basin [10]. In Algeria, *P. lentiscus* is dispersed along the entire littoral [11] and grows in diverse habitats along a climatic gradient that varies in solar radiation, temperature, and precipitation. 

*P. lentiscus* is extensively used in folk medicine [12], and the pharmaceutical and antimicrobial activity of this species has been reported by several authors [13,14]. The Anacardiaceae is a family that secrete substances containing terpenes and carbohydrates in ducts which are found in vascular rays, especially from the genus *Pistacia* [15]. *P. lentiscus* is recognized as a terpene-storing species which produces the largest number of individual terpenes [16].

Numerous essential oil studies have been conducted on *P. lentiscus* leaves from different provenances [17,18,19,20,21] but, neither the plant sex, which is an additional source of variability [22] nor the ecological conditions of plant growth were taken into account. Because of the differential volatility of terpenes, this prompted us to study the variability of terpenoid content of female *P. lentiscus* with increasing altitude. The result of this work will allow us to further understand the ecology of the plant in a constrained environment given its importance in reforestation programs in semi-arid Mediterranean Regions.

## 2. Results and Discussion

The leaves of *P. lentiscus* contain 49 identified compounds, which are listed in Table 1. Among these, twelve are monoterpenes (eight hydrocarbons, two oxygenated and two derivatives) and thirty seven are sesquiterpenes (25 hydrocarbons and 12 oxygenated). 

**Table 1:** Concentration of terpenoids (µg g^–1^ dw) found in female *Pistacia lentiscus* L. leaves from low, mid and high altitude sites in Algeria.

Compounds	Mean concentration ± standard deviation of terpenoid contents in leaves extracts of *Pistacia lentiscus* (µg.g dw^−1^)															
	RI	Low altitude					Mid altitude					High altitude				
		L1(n = 5)	L2(n = 5)	L3(n = 5)	L4(n = 5)	L5(n = 5)	M1(n = 5)	M2(n = 5)	M3(n = 5)	M4(n = 5)	M5(n = 5)	H1(n = 5)	H2(n = 5)	H3(n = 5)	H4(n = 5)	H5(n = 5)
α-thujene	926	4.8±1.6	3.8±1.3	1.1±0.9	1.4±0.9	4.6±1.5	8.2±1.1	8.1±0.4	4.5±0.9	3.4±0.5	6.5±1.2	13.6±0.7	15.2±0.7	12.5±1.5	15.1±0.7	16.9±0.5
α-pinene	933	8.1±3.3	7.6±3.0	2.9±1.1	2.7±0.9	5.5±1.2	2.2±0.2	5.7±0.9	5.2±0.8	1.9±0.3	13.5±2.0	**102.9±** **6.2**	**100.9±** **3.6**	**76.3±** **14.2**	**105.0±** **4.5**	**98.0±** **0.5**
camphene	947	0.6±0.2	0.6±0.2	0.3±0.1	0.3±0.1	0.4±0.1	0.9±0.2	3.0±1.1	1.1±0.5	0.9±0.3	2.2±0.7	14.3±1.3	17.1±1.8	12.8±3.4	15.9±2.1	20.3±0.6
β-pinene	973	12.2±4.3	10.9±3.8	3.0±1.5	3.3±1.5	7.9±2.3	11.5±1.3	12.1±1.8	11.4±3.0	4.5±0.4	14.5±2.2	35.3±7.4	36.7±7.1	56.2±14.9	44.3±8.8	30.9±0.6
α-terpinene	1018	2.0±0.9	1.9±0.7	0.1±0.1	0.6±0.3	1.9±0.6	7.3±0.7	6.2±1.4	2.9±1.1	1.3±0.3	1.9±0.4	7.9±0.6	9.3±0.5	8.5±1.4	9.5±0.4	10.5±0.4
p-cymene	1027	0.9±0.4	0.9±0.4	0.4±0.1	0.3±0.1	0.7±0.1	1.9±0.4	3.5±0.9	1.4±0.5	0.7±0.3	1.6±0.6	15.7±0.7	17.7±0.5	21.3±4.8	18.5±0.5	18.8±0.4
limonene	1029	2.3±0.2	2.3±0.3	1.7±0.0	1.5±0.1	2.1±0.1	5.8±0.5	5.7±1.6	2.9±0.9	2.4±0.3	4.0±0.4	11.5±2.6	13.6±2.2	32.6±12.3	16.3±2.7	12.7±0.3
γ-terpinene	1062	2.1±0.6	1.7±0.6	0.5±0.3	0.8±0.4	2.0±0.6	4.7±0.4	5.6±1.0	2.5±0.8	1.4±0.3	2.4±0.4	7.2±0.6	8.9±0.7	7.7±0.9	8.8±0.7	10.5±0.4
terpinen-4-ol	1179	5.1±1.2	4.0±0.7	3.5±0.6	2.4±0.1	3.1±0.4	5.9±0.8	7.0±1.3	4.6±0.9	4.1±0.4	6.3±0.6	27.4±0.6	29.6±0.4	42.5±7.8	30.3±0.4	30.5±0.4
α-terpineol	1197	2.4±0.6	1.6±0.5	0.8±0.2	0.5±0.2	1.4±0.1	1.9±0.3	3.5±1.0	2.0±0.5	1.1±0.3	2.0±0.7	5.1±0.8	7.5±0.5	6.0±1.1	8.4±0.6	8.2±0.4
borneol acetate	1287	6.8±1.9	5.6±1.5	3.9±0.2	3.6±0.2	3.2±0.4	7.3±0.8	6.5±1.1	6.3±1.5	6.9±0.7	18.7±6.2	18.6±0.7	22.3±0.5	25.8±6.9	22.4±0.5	23.6±0.4
α-terpineol acetate*	1351	4.4±0.2	4.0±0.6	10.3±1.0	8.8±0.5	8.9±2.9	17.7±2.3	10.4±1.5	5.2±1.4	7.9±0.4	14.1±3.8	15.3±0.3	16.5±0.2	11.7±1.5	16.7±0.2	17.2±0.2
δ-elemene*	1338	0.5±0.1	0.5±0.1	0.6±0.1	0.4±0.0	0.0±0.0	1.2±0.3	2.8±0.9	0.7±0.4	1.7±0.3	7.0±3.1	0.4±0.2	2.6±0.5	1.4±0.9	2.7±0.4	3.9±0.4
α-cubebene*	1351	4.4±0.2	4.0±0.6	10.3±1.0	8.8±0.5	8.9±2.9	17.7±2.3	10.4±1.5	5.2±1.4	7.9±0.4	14.1±3.8	15.3±0.3	16.5±0.2	11.7±1.5	16.7±0.2	17.2±0.2
α-copaene	1377	7.5±1.3	8.3±1.0	10.4±1.1	8.5±0.6	13.7±3.1	22.9±3.7	10.9±0.8	9.4±0.3	8.8±0.5	13.9±1.0	34.8±2.4	38.3±1.5	23.2±4.7	40.3±1.9	37.8±0.4
β-bourbonene*	1389	1.7±0.2	1.2±0.1	0.7±0.1	0.7±0.1	0.8±0.3	1.3±0.3	3.0±0.9	1.3±0.3	0.8±0.3	5.1±1.2	7.2±0.9	9.9±1.3	2.4±0.3	6.0±1.2	12.2±0.4
β-cubebene*	1391	3.9±0.5	3.3±0.5	14.2±1.0	13.8±1.3	32.5±8.7	21.0±3.6	10.4±2.0	4.5±1.1	11.0±0.9	5.7±1.1	13.2±0.6	16.4±0.5	9.6±2.0	16.7±0.4	17.7±0.4
β-elemene*	1393	34.9±0.9	34.4±0.4	3.1±0.4	2.5±0.3	3.4±0.5	4.4±0.7	5.5±0.5	3.9±0.6	3.5±0.3	7.3±0.6	4.5±1.2	6.9±0.8	6.7±1.8	8.2±1.0	7.2±0.4
β-caryophyllene	1422	92.7±18.5	78.4±16.4	71.4±6.8	54.3±2.7	57.2±4.7	95.5±6.6	71.4±2.9	80.3±14.9	82.8±9.0	146.8±10.8	178.2±14.9	186.3±11.7	156.4±30.1	198.5±14.3	175.6±0.4
β-copaene*	1431	6.6±0.6	6.2±0.5	6.0±0.5	5.0±0.2	17.3±11.6	10.4±1.5	7.6±0.8	5.9±0.6	7.1±1.0	9.4±1.0	14.7±4.5	17.2±3.9	13.2±2.4	21.6±4.9	14.3±0.4
γ-elemene*	1440	0.5±0.1	0.4±0.1	0.2±0.1	0.2±0.1	0.4±0.1	1.7±0.3	3.4±1.1	1.0±0.5	0.7±0.3	0.8±0.3	2.3±0.2	4.4±0.6	2.4±0.4	4.4±0.6	5.9±0.4
*trans*-muurola-3,5-diene*	1451	2.2±0.6	3.3±0.7	6.4±0.8	5.9±0.8	4.4±0.9	7.4±1.0	8.3±1.6	3.6±0.8	4.9±0.2	5.3±1.0	2.9±0.6	4.4±0.9	2.8±0.4	3.8±1.1	6.3±0.3
α-humulene	1457	20.9±2.6	19.3±3.0	20.5±1.7	16.3±0.7	19.4±1.8	35.6±4.9	19.1±0.7	18.6±2.1	23.9±1.8	30.8±1.6	28.4±6.6	33.3±7.6	29.0±5.6	26.2±9.3	42.0±0.4
allo-aromadendrene*	1462	6.4±0.5	5.6±0.7	7.6±0.7	6.1±0.3	8.0±0.9	17.5±2.6	9.2±1.2	5.9±0.8	7.5±0.4	9.3±0.9	15.8±3.2	18.7±2.6	10.2±2.2	21.8±3.3	17.1±0.4
*cis*-muurola-4(14),5-diene*	1465	3.8±0.3	3.6±0.3	3.7±0.3	3.5±0.7	3.9±0.6	5.9±0.9	5.3±0.7	3.5±0.3	4.6±0.4	5.4±0.6	7.0±2.4	9.7±1.8	6.6±1.1	12.0±2.3	8.8±0.4
*trans*-cadina-1(6),4-diene*	1476	2.7±0.3	2.8±0.5	6.8±0.5	5.0±0.3	5.5±0.6	3.8±0.7	5.9±1.3	3.2±0.7	5.0±0.6	3.2±0.7	12.4±1.2	15.4±0.6	5.7±1.3	16.4±0.7	15.9±0.4
γ-muurolene*	1479	32.5±1.8	29.7±3.1	37.3±1.9	30.8±1.0	34.7±4.0	62.5±6.7	36.7±1.9	28.6±2.4	29.9±2.9	37.7±2.4	59.7±5.4	62.6±4.4	40.7±5.9	67.3±5.4	58.9±0.2
germacrene D*	1484	23.5±3.3	21.5±2.0	16.4±0.8	13.9±0.5	14.1±0.8	19.0±1.1	19.3±2.4	19.0±2.4	24.3±6.8	37.6±5.0	33.7±14.7	36.9±13.9	44.6±9.3	51.4±17.1	24.0±0.4
β-selinene*	1489	7.5±0.8	7.1±0.5	5.7±0.7	5.4±0.2	8.4±0.8	10.7±1.2	8.9±0.7	6.1±0.6	6.0±0.3	9.7±1.1	8.7±2.6	11.3±2.1	10.0±1.6	13.8±2.6	10.2±0.4
*trans*-muurola-1(14),5-diene*	1494	5.5±0.4	5.8±0.9	13.6±0.8	10.6±0.5	13.1±0.9	14.7±2.9	6.8±0.5	4.7±0.2	10.5±0.8	7.6±1.0	15.3±1.4	18.3±0.8	9.7±2.1	19.5±0.9	18.6±0.4
α-muurolene*	1503	16.1±1.6	17.1±2.3	27.6±2.3	21.9±1.4	25.7±2.9	46.8±5.9	24.7±1.9	16.4±2.5	26.1±1.2	24.3±2.1	42.9±5.2	55.7±0.9	24.6±5.2	57.1±1.1	55.9±0.4
β-bisabolene*	1510	22.1±2.3	21.6±2.5	81.9±5.4	62.5±5.3	74.3±8.2	105.0±19.9	38.5±9.2	21.4±2.3	63.6±4.0	36.6±4.8	55.3±5.9	60.0±4.5	42.7±8.6	65.1±5.6	56.5±0.4
γ-cadinene*	1517	15.7±1.2	15.3±1.0	12.6±1.3	11.7±0.8	23.7±9.9	32.8±2.9	35.4±8.0	20.2±6.6	15.3±0.7	20.5±2.0	24.9±8.3	27.0±7.9	19.9±3.4	35.4±9.7	20.2±0.4
δ-cadinene*	1528	60.3±5.5	59.0±9.3	112.5±9.5	83.3±3.8	98.4±9.1	176.2±19.6	88.4±8.5	59.7±10.7	98.6±4.8	83.9±8.0	143.6±19.0	153.9±16.3	87.4±18.2	170.7±20.0	138.6±0.4
cadina-1,4-diene*	1535	7.1±0.8	5.4±0.6	6.3±0.5	6.1±0.6	9.8±1.2	11.2±0.5	10.4±2.0	5.2±1.6	5.0±0.5	3.9±2.3	7.1±0.5	8.4±0.4	7.1±0.5	8.8±0.3	9.5±0.4
α-cadinene*	1541	4.9±0.4	4.1±0.3	14.1±0.4	13.1±0.1	5.1±0.5	10.4±1.7	14.6±4.0	7.0±3.0	5.1±0.5	5.4±0.8	6.6±1.6	8.2±1.3	5.0±0.8	10.0±1.6	8.0±0.4
α-calacorene*	1546	1.2±0.1	1.2±0.0	1.2±0.1	1.3±0.0	1.3±0.1	7.9±0.5	8.2±0.4	8.7±0.8	3.5±0.4	4.1±0.2	1.7±0.2	1.3±0.0	1.2±0.1	1.3±0.0	1.3±0.1
cubebol*	1520	15.8±3.5	15.5±3.0	112.9±12.0	75.5±20.6	108.9±16.4	117.6±21.7	60.3±27.8	17.9±4.9	71.4±5.0	36.6±7.8	57.8±2.5	62.3±1.6	42.4±9.3	61.3±1.8	64.8±0.4
elemol*	1553	8.7±2.0	8.4±1.4	7.5±1.2	6.3±1.0	9.4±1.8	6.7±0.7	10.3±1.5	5.9±1.2	7.8±0.3	11.5±0.8	9.6±1.5	13.1±0.7	9.6±1.9	14.3±0.9	13.5±0.4
germacrene D-4-ol*	1584	1.2±0.0	1.2±0.0	1.1±0.1	1.2±0.1	1.1±0.1	1.8±0.3	3.6±1.0	1.6±0.4	1.1±0.1	1.0±0.3	1.1±0.0	1.1±0.1	1.2±0.0	1.1±0.1	1.6±0.1
spathulenol*	1584	13.0±2.8	11.8±2.2	9.7±1.1	11.9±0.5	18.5±4.4	10.1±0.9	13.2±1.6	10.8±2.6	7.6±0.7	40.6±18.0	13.1±1.2	26.7±11.0	22.4±3.6	28.0±10.7	38.2±13.5
caryophyllene oxide	1587	56.0±18.3	52.0±15.9	53.0±8.6	45.6±6.4	67.7±11.6	54.8±2.2	53.3±2.1	55.1±14.3	48.1±1.7	71.8±6.6	58.6±2.6	70.4±10.2	49.1±9.0	67.9±11.0	83.6±10.9
gleenol*	1588	1.2±0.0	1.2±0.0	1.1±0.1	1.2±0.1	1.1±0.1	9.8±0.3	11.7±1.0	3.2±2.0	1.1±0.1	11.2±4.1	1.1±0.0	1.1±0.1	1.2±0.0	1.1±0.1	1.6±0.1
humulene oxide II	1613	12.3±2.0	11.5±1.7	16.8±1.9	15.2±1.5	24.3±3.0	22.6±3.2	13.5±1.2	11.1±1.5	16.0±1.2	14.7±1.5	17.6±2.4	21.3±2.3	12.7±2.6	23.7±2.2	22.4±2.1
1-epi-cubenol*	1632	6.0±3.3	5.9±2.9	16.3±1.3	12.3±1.3	10.4±0.9	14.1±2.2	10.9±1.7	6.8±2.4	12.5±1.8	11.6±2.5	9.9±0.8	12.4±0.5	4.0±1.1	13.0±0.4	13.4±0.5
α-muurolol*	1648	11.5±2.3	11.7±2.1	26.9±1.5	23.0±1.6	20.3±5.1	19.0±2.5	14.8±1.2	10.4±2.1	21.6±1.2	12.3±2.4	15.9±3.6	21.3±3.8	13.8±2.8	24.7±3.8	22.2±3.8
α-cadinol*	1661	15.1±2.8	17.4±1.9	22.7±0.8	21.4±1.0	24.6±1.3	21.0±1.4	29.6±0.9	18.2±3.2	24.8±0.7	19.5±2.8	17.9±5.6	24.0±5.7	18.6±2.3	29.4±5.9	23.9±5.0
α-bisabolol*	1688	9.9±0.6	9.8±0.5	10.7±0.5	10.3±0.4	10.3±0.5	32.8±0.9	34.0±2.0	15.5±5.8	27.3±0.8	15.1±6.9	4.4±0.3	5.8±0.4	5.8±0.5	7.6±0.6	7.2±0.2
eudesma-4(15),7-dien-1-beta-ol*	1696	10.5±2.9	6.9±0.7	22.8±2.6	19.5±1.5	27.5±3.4	26.0±0.5	35.3±2.5	21.3±3.1	11.3±3.4	27.5±2.1	8.8±1.0	11.9±1.1	7.5±1.1	11.4±1.2	13.8±0.5
**Total** **monoterpenes**	*39±* *3.9*						*65±* *5.0*					*299±* *6.8*				
*Total monoterpene hydrocarbons*	*21.7±* *3.4*						*37.5±3.2*					*221.5±5.3*				
*Total oxygenated monoterpenes*	*16.8±1.9*						*27.8±2.4*					*77.1±2.5*				
*Total sesquiterpenes*	*799±38.0*						*956±48.6*					*1181±55.0*				
*Total sesquiterpene hydrocarbons*	*422.18±18.0*						*509.1±32.4*					*762.5±37.1*				
*Total oxygenated sesquiterpenes*	*377.2±22.7*						*446.9±20.7*					*418.9±23.3*				
*Total terpenes*	*838±39.0*						*1021±51.6*					*1480±57.7*				

RI: Retention Index; NI: Non Identified. ; * : tentatively identified.

For a given altitude level five populations were selected, each of them exhibiting a low intra-populational variability regarding the individual components identified. However for low and mid-altitudes inter-population variability increases. For instance β-elemene content in lowlands plants ranged from 2.5–34.9 µg g^−1^ dw. This raises the complexity of the identification of molecular markers that discriminate ecological gradients. Taking into account such variability the main compounds found in low altitude sites were: δ-cadinene (50–112 µg g^−1^ dw), β-caryophyllene (54–92 µg g^-1^ dw) and cubebol (15–112 µg g^−1^ dw). Mid-altitude site samples contained similar main components: δ-cadinene (59–176 µg g^−1^ dw), β-caryophyllene (71–146 µg g^−1^ dw) and cubebol (17–117 µg g^−1^ dw). However, in high altitude site, β-caryophyllene (156–186 µg g^−1^ dw), δ-cadinene (83–153 µg g^−1^ dw) and α-pinene (76–105 µg g^−1^ dw) were the dominant components. Hence, the three altitudinal levels were qualitatively similar except for the high content of α-pinene found in highlands specimens. When considering the amount of the different families of terpenic constituents, Two-Way-ANOVA showed that there is no significant effect of the interaction between site and station for monoterpene hydrocarbons (Table 2). The subsequent One-Way-ANOVA carried out followed by a Tukey’s post hoc test showed that monoterpene hydrocarbons found in low (21.7 µg g^−1^ dw), mid- (37.5 µg g^−1^ dw) and highlands (221.5 µg g^−1^ dw) were significantly different (F = 742.22; p < 0.001). Thus, we may conclude that monoterpene hydrocarbons significantly increase with altitude. The high richness of terpenes content found in leaves of *P. lentiscus* of the high elevated site is in accordance with several authors who have stated that altitude is a factor influencing plant chemistry [23,24]. Enhanced UV-B radiation and lower temperatures at high altitudes have been exhaustively discussed as having an impact on flavonoid and monoterpene hydrocarbon contents [25].

**Table 2:** Variance analysis (Two-Way Anovatest, 5%) of monoterpenes (hydrocarbons and oxygenated), sesquiterpenes (hydrocarbons and oxygenated) and total terpenes between sites, stations and interaction site *vs* station.

Terpenoids	factors	df	SS	MS	F	*P*
*Monoterpene hydrocarbons*	Site	2	617137	308569	479.144	< 0.001
	Site:Station	12	7725	644	1.754	0.08
	Residuals	60	22007	367		
*Oxygenated monoterpenes*	Site	2	51551	25775.3	108.802	< 0.001
	Site:Station	12	2843	236.9	2.823	0.004
	Residuals	60	5037	83.9		
*Sesquiterpene hydrocarbons*	Site	2	1563493	781746	11.700	0.001
	Site:Station	12	801748	66812	4.718	< 0.001
	Residuals	60	849581	14160		
*Oxygenated sesquiterpenes*	Site	2	61397	30698	0.688	0.52
	Site:Station	12	535103	44592	7.484	< 0.001
	Residuals	60	357500	5958		
*Total terpenes*	Site	2	5470660	2735330	13.782	< 0.001
	Site:Station	12	2381658	1984472	5.601	< 0.001
	Residuals	60	2126026	35434		

**Figure 1:**
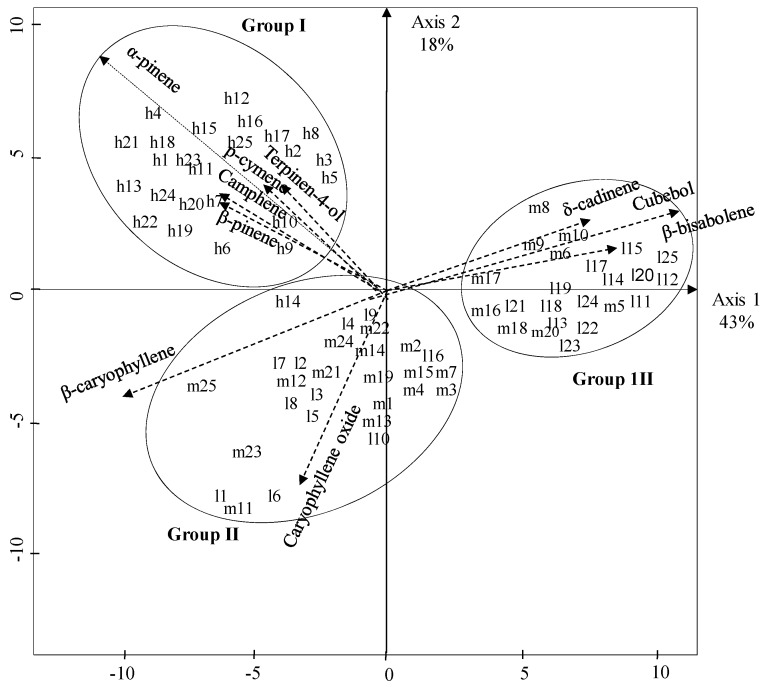
Tow-dimensional mapping of the P.C.A. analysis of *Pistacia lentiscus* L*.* individual distribution from low, mid and high altitude sites in Algeria (l: low; m: medium and h: high altitude sites).

Because the individual oil components, expressed on dw basis (w/w) could not clearly discriminate between the three altitude levels, data were expressed in terms of percentage of the chromatogram prior to Principal Component Analysis (PCA). Figure 1 represents two-dimensional mapping of the PCA, Axis 1 and Axis 2 represent 43% and 18% of the information, respectively. 

The distribution of individual points in plan 1-2 based on cluster analysis (Ward’s technique) divided the 75 accessions into three main groups according to their content (expressed in % because of maximum of variability) in α-pinene, caryophyllene oxide and cubebol. The group I (33.3% of total samples) includes individuals harvested from the high elevated sites which are characterized by a high content in β-caryophyllene (12.0%), δ-cadinene (9.3%) and α-pinene (6.8%). Among monoterpenes which characterize this group, camphene, p-cymene, β-pinene, terpinen-4-ol are also the most discriminating compounds. The groups II and III are dominated by individuals harvested from low and mid-altitude sites, respectively. The chemical composition of group II (37.3% of samples) was characterized by high contents of β-caryophyllene (11.5%), δ-cadinene (8.6%) and caryophyllene oxide (6.8%). The last group (26.4% of samples) contains mainly individuals of low altitude sites with δ-cadinene (10.9%), cubebol (10.5%) and β-bisabolene (7.7%) as discriminating components. Samples of Groups I and II were similar to Sardinian (Costa Rey) chemotype where β-caryophyllene (31.38%), germacrene D (12.08%) and δ-cadinene (6.48%) were identified as the dominant compounds from supercritical CO_2_ leaves extracts [26]. However the composition of the hydrodistillates were totally different. The chemical variability of *P. lentiscus* essential oil obtained through hydrodistillation from samples harvested at Tigzirt (Algeria) was reported as α-pinene (22–29%), myrcene (1.4–23%) and sabinene (8–11.7%) were the major constituents [27]. In our samples from Tigzirt (see Table 1: L3) myrcene and sabinene were not detected. This disagreement with our data is probably the result of thermal hydrolysis that occurs during hydrodistillation, as suggested earlier [26]. Consequently, it is likely that cold extraction leads to the recovery of the true components in plants. That said, axis 1 clearly separates Groups I and III while axis 2 distinguishes Group I from Group II. This raises the question of the characterization of the ecological factors involved. A careful analysis of our sampling sites (Table 3) revealed that lowlands habitats were highly heterogeneous in terms of specific composition. All the highland habitats were composed by *Olea europea* plant association. Taking into account the other ecological data collected, the mean maximum temperature in highlands (about 27 °C) was lower that that of the lowlands (31 °C) suggesting that, axis 1 determines temperatures decrease. Because most of lowlands were located near the sea, it is not excluded that other factors such as substrate type and soil salinity may be particularly discriminating factors. On the other hand axis 2 can be interpreted as the result of abiotic factors associated with drought since midlands exhibited a lower mean coefficient of Emberger (Q_2_ = 85) compared to that of highlands (Q_2_ = 154).

Among the identified components, α-pinene, camphene, β-pinene and p-cymene are the most discriminating monoterpene hydrocarbons of individuals from high altitude sites. The increase of these compounds with altitude regardless to the specific composition of the habitats may be explained by the involvement of genetic and/or abiotic factors. For instance camphene is found to be cytoprotective by decreasing lipid peroxidation and inhibiting NO release and ROS generation [28] whereas, p-cymene was reported to be positively correlated with aridity index and altitude from *Thymus piperella* [29].

## 3. Experimental

### 3.1. Plant material

Seventy five sample of *Pistacia lentiscus* were collected in September 2008 in three Algerian sites which are located at different altitudes. Five stations were chosen per site with five female individuals per station. The first site includes individuals harvested from low altitude, the second one includes individuals located at mid-altitude and finally, we have collected individuals at the high altitude site in Kabylia. Ecological factors of the sampling sites are described in Table 3.

Sampling was carried out during fructification stage in order to take into account the phenological shift due to local climatic conditions. Twenty five female individuals were chosen per site. Mature and sun exposed leaves were harvested, dried in dark at ambient air temperature (25 °C) conditions until constant weight then, 100 g per tree were grounded and stored until use. 

### 3.2. Terpenoids extraction

The extraction method used consisted of suspending leaf dry matter in dichloromethane according to a ratio of 1:2 (w/v), for 30 min, under constant shaking at room temperature. Dodecane (50 µL, 5 mg mL^−1^) were added as internal standard for quantification.

**Table 3:** Ecological factors of *Pistacia lentiscus* collection sites.

Sites	Stations	Alt.(m)	Geographical coordinates	Habitat	P (mm)	T^0 ^Max (C^0^)	Q_2_
Low-altitude	Cherchel (L1)	15	36°36’N/2°11’E	*Phillyrea latifolia*	502.24±25.08	31.75±0.42	73.54
	Azzefoun (L2)	14	36°53’N/4°25’E	*Phillyrea latifolia*	775.78±50.04	31.50±0.48	122.06
	Zemmouri Bahri (L5)	9	36°48’N/3°35’E	*Erica arborea*	741.14±45.34	29.10±0.51	125.85
	Tigzirt (L3)	6	36°53’N/4°08’E	*Arbutus unedo*	923.09±67.98	30.30±0.44	150.77
	Ziama Mansouriah (L4)	8	36°39^’^N/5°28’E	*Erica arborea*	968.11±60.82	30.80±0.38	148.24
Mid-altitude	Thenia (M1)	168	36°43’N/3°32’E	*Eucalyptus radiata*	728.53±58.43	35.00±0.37	88.61
	Beni Amrane (M4)	121	36°39’N/5°14’E	*Erica arborea*	729.58±55.22	35.50±0.38	87.50
	Tizi Ouzou (M5)	182	36°43’N/4°03’E	*Pinus halepensis*	731.92±57.42	36.10±0.41	83.68
	El Kseur (M3)	167	36°41’N/4°51’E	*Olea europea*	758.39±59.33	30.80±0.35	112.12
	Lakhdaria (M2)	307	36°34’N/3°35’E	*Olea europea*	513.85±54.14	35.60±0.39	55.25
High- altitude	Ait Khelifa (H3)	1042	36°31’N/4°20’E	*Olea europea*	1039.30±56.57	23.20±0.32	168.95
	Tala Moumene (H4)	1008	36°33’N/4°48’E	*Olea europea*	1041.27±67.45	24.46±0.35	190.18
	Beni Mendes (H1)	809	36°30’N/3°59’E	*Olea europea*	924.56±64.54	31.75±0.38	112.74
	Aourir N’Athjelil (H2)	868	36°34’N/4°48’E	*Olea europea*	1019.62±69.33	25.44±0.31	182.15
	Tizi-Oumalou (H5)	942	36°30’N/4°20’E	*Olea europea*	939.06±78.75	30.82±0.36	116.11

*Data sources*: Office Nationale de la Météorologie d’Alger (O.N.M.) and Agence Nationale des Ressources Hydriques d’Alger (A.N.R.H.). Period from 1997–2007; Specimens were deposited at the herbarium of the University of Provence (Marseille) and referred as PL1-MAR -2010, PL2-MAR-2010, PL3-MAR-2010 for Algerian low, mid and high altitude sites, respectively.

### 3.3. Quantitative and qualitative analysis of terpenoids

Extracts were filtered on an Regenerated Cellulose syringe filter (RC, 0.45 µm, 25 mm, Phenomenex) then analyzed with a gas chromatograph (Hewlett Packard® GC 6890) coupled to a mass selective detector (5973 Network). The system was fitted with an HP-5MS capillary column (30 m, 0.25 mm, 0.25 µm). Extract (2 µL) was injected through an automatic injector (ALS 7683) in splitless mode. Purge was set at 50 min mL^−1^ after 1 min. Injection temperature was maintained at 250 °C. Helium was used as carrier gas. A constant flow rate of 1 mL min^-1^ was set throughout the run. The oven temperature initially set at 40 °C was increased to 270 °C at a rate of 4 °C min^−1^ and remained constant for 5 min. The MSD transfer line heater was maintained at 280 °C. Terpenes were identified by comparison of their retention index (RI) and mass spectra (NIST, 2008) with those obtained from authentic samples and literature [30].

### 3.4. Statistical analyses

The data were analyzed by a two-way ANOVA model. Turkey’s HSD (Honestly Significant Differences) procedure was used to test for significant differences in monoterpenes (hydrocarbons and oxygenated), sesquiterpenes (hydrocarbons and oxygenated) and total terpene concentrations between stations and sites. In order to evaluate the qualitative distribution of major chemical compounds (expressed in % of the chromatogram) in our 75 samples, Principal Component Analysis (PCA) was carried out. The statistical analyses were performed using the R statistical software [31], packages "ade4" [32] and “agricolae” [33].

## 4. Conclusions

Our investigation revealed a chemical diversity of *P. lentiscus* associated with the geographic location*.* Three chemotypes were identified according to the different elevation levels. This variability may be interpreted as the result of biotic and abiotic factors, as well as genetic variability. The main discriminating environmental factors identified were temperature and drought. Consequently our finding should be further verified through analysis of terpenoids in seedlings of multiple provenance held under identical growth conditions. 
